# Uses and Misuses of Recorded Mental Health Lived Experience Narratives in Healthcare and Community Settings: Systematic Review

**DOI:** 10.1093/schbul/sbab097

**Published:** 2021-08-23

**Authors:** Caroline Yeo, Stefan Rennick-Egglestone, Victoria Armstrong, Marit Borg, Donna Franklin, Trude Klevan, Joy Llewellyn-Beardsley, Christopher Newby, Fiona Ng, Naomi Thorpe, Jijian Voronka, Mike Slade

**Affiliations:** School of Health Sciences, Institute of Mental Health, University of Nottingham, Nottingham, UK; School of Health Sciences, Institute of Mental Health, University of Nottingham, Nottingham, UK; Disability North, Newcastle, UK; Center for Mental Health and Substance Abuse, Faculty of Health and Social Sciences, University of South-Eastern Norway, Drammen, Norway; NEON Lived Experience Advisory Panel, Nottingham, UK; Center for Mental Health and Substance Abuse, Faculty of Health and Social Sciences, University of South-Eastern Norway, Drammen, Norway; School of Health Sciences, Institute of Mental Health, University of Nottingham, Nottingham, UK; School of Medicine, Institute of Mental Health, University of Nottingham, Nottingham, UK; School of Health Sciences, Institute of Mental Health, University of Nottingham, Nottingham, UK; Nottinghamshire Healthcare NHS Foundation Trust, Library and Knowledge Services, Duncan Macmillan House Staff Library, Nottingham, UK; School of Social Work, University of Windsor, Windsor, Canada; School of Health Sciences, Institute of Mental Health, University of Nottingham, Nottingham, UK

**Keywords:** recovery story, testimony, madness, critique, psychosis, autobiography

## Abstract

Mental health lived experience narratives are first-person accounts of people with experience of mental health problems. They have been published in journals, books and online, and used in healthcare interventions and anti-stigma campaigns. There are concerns about their potential misuse. A four-language systematic review was conducted of published literature characterizing uses and misuses of mental health lived experience narratives within healthcare and community settings. 6531 documents in four languages (English, Danish, Swedish, Norwegian) were screened and 78 documents from 11 countries were included. Twenty-seven uses were identified in five categories: political, societal, community, service level and individual. Eleven misuses were found, categorized as relating to the narrative (narratives may be co-opted, narratives may be used against the author, narratives may be used for different purpose than authorial intent, narratives may be reinterpreted by others, narratives may become patient porn, narratives may lack diversity), relating to the narrator (narrator may be subject to unethical editing practises, narrator may be subject to coercion, narrator may be harmed) and relating to the audience (audience may be triggered, audience may misunderstand). Four open questions were identified: does including a researcher’s personal mental health narrative reduce the credibility of their research?: should the confidentiality of narrators be protected?; who should profit from narratives?; how reliable are narratives as evidence?)

## Introduction

Mental health lived experience narratives are first-person accounts of people with experience of mental health problems. They can be shared with others, either live as part of a relationship, or recorded via text, audio or video.^1^ Live narratives can be told in the context of a peer support work relationship^2^ and as a component of the training^3^ of health and social care clinicians. Narratives can be published as a memoir^4^ or in a collection of narratives.^5^ Narratives are also compiled and published in collections by mental health services^6^ or groups of people with lived experience and activists such as Recovery Devon.^7^

Digital technology has made narratives available wide scale on the internet through video platforms such as YouTube. National anti-stigma campaigns such as Time to Change have used lived experience narratives in their campaign to address community attitudes.^8^ They are also used in digital interventions such as in Narrative Experiences Online (NEON) Intervention^9^ currently being evaluated in three clinical trials.^10^ Lived experience narratives have also played a role in mental health research activities as data for analysis or in the case of digital storytelling as a potential research method for facilitating shared dialogue between stakeholders in mental health on lived experience.^11^

Lived experience narratives have been collected and shared by some academic journals to provide readers with insights into the phenomenology of mental health problems. They have been published in academic journals such as the *Journal of Psychiatric and Mental Health Nursing*,^12^*Mental Health and Social Inclusion*,^13^ and *Schizophrenia Bulletin* has a long-running First Person Accounts series, which has published since 1979.^14^

Lived experience narratives are presented in ways which are idiosyncratic to their narrators, and while some narrative might make use of biomedical concepts such as diagnosis, others might draw on alternative conceptualizations such as spiritual experiences^15^ or mental health distress caused by structural or social issues such as racism.^16^

The subset of lived experience narratives which describe recovery from mental health problems has been extensively studied.^17^ A systematic review has defined a recovery narrative as a story told by a person about their journey of recovery, which includes elements of both adversity or struggle, and of strength, success, or survival related, at least in part, to mental health problems and which refer to events or actions over a period of time.^18^ The focus of the current review is on the wider lived experience narrative since the recovery narrative definition may limit the diversity of experiences mental health distress that exist.^19^

Lived experience narratives have been considered at the heart of the psychiatric survivor movement for organizing resistance and change, but there is growing concern about the possible misuse of them,^20^ and hence the possibility of causing harm to narrators or others. An example is the co-option of narratives to promote neoliberal agendas.^3^ Co-option is a “process by which a dominant group attempts to absorb or neutralize a weaker opposition that it believes poses a threat to its continued power.” ^21^ Co-option can harm a narrator if their story becomes a commodity to be used by others to promote conflicting agendas.^3^

The VOICES typology^17,22^ listed 13 purposes for lived experience narratives as described by curators (those who bring together, edit and organize collections of narratives). These included “building a narrative collection to act as an evidence base for rights movement” and “facilitating emancipatory action.” ^17^

While it is broadly accepted that lived experience narratives are used for specific purposes, no systematic review has been conducted on their usage in a health service or community setting. The closest available evidence is a systematic review on the educational uses of lived experience narratives.^23^

The aim of the review is to investigate uses and misuses of recorded mental health lived experience narratives in healthcare and community settings.

The objectives of this work are: (1) to identify actual and proposed uses of recorded mental health lived experience narratives; (2) to synthesize critiques about their actual or potential misuse; and (3) to make recommendations for best practice in using recorded mental health lived experience narratives.

## Method

Preferred Reporting Items for Systematic Reviews and Meta-Analyses (PRISMA) guidance on systematic reviews was followed.^24^ The review protocol was pre-registered with the International Prospective Register of Systematic Reviews PROSPERO 2021 and can be found at: https://www.crd.york.ac.uk/prospero/display_record.php?ID=CRD42021229458.

### Inclusion Criteria

Lived experience narratives were defined as: first person accounts that refer to events or actions over time and which include elements of adversity or struggle where adversity or struggle relate to mental health problems. This was adapted from the narrower definition of a recovery narrative used in a prior systematic review.^18^ Recorded narratives are those presented in an invariant form, such as text, audio or video. Misuses were defined as concerns or critiques about actual or potential misuses of narratives.

Inclusion criteria were: describes actual or potential use of recorded mental health lived experience narrative/collections OR presents specific misuse of actual or proposed use (as judged by the review team); relates to a healthcare or community setting; full text is available in English, Danish, Swedish or Norwegian (Bokmål or Nynorsk).

Systematic reviews were excluded unless they synthesized evidence from studies to generate a critique, in which case only critique was considered in-scope for synthesis. Healthcare settings included any setting operated or controlled by a state or private health care service, including primary, secondary, tertiary, and palliative care. Community settings included community organizations such as not-for-profit organizations, charities and activism groups. Research in educational settings such as schools and recovery colleges was excluded since a prior review^23^ had considered this.

### Search Strategy

#### Database Searches

The search strategy was developed with an Information Specialist (NT). The following databases were searched in English: Applied and Complementary Medicine (AMED), MEDLINE, PsycINFO via Ovid; Applied Social Science Index and Abstracts via ProQuest; Cumulative Index of Nursing and Applied Health Literature (CINAHL) via EBSCOhost Research Databases; Scopus via Elsevier; Arts and Humanities Citation Index and Social Science Citation Index, via Web of Science. The search terms used in the PsycINFO search, identified from a combination of free text terms (title and abstract), relevant controlled vocabulary headings, and advanced searching syntax. The search terms used in the PsycINFO search, identified from the title or abstract of documents are found in online supplement 1.

The keyword and, where relevant, subject heading searches were subsequently tailored to each database.

Scandinavian databases searched with translated terms comprise: Idunn^25^ is a Scandinavian digital publishing platform for academic journals and books and Oria^26^ is a search engines for Norwegian academic libraries’ resources; SveMed+ ^27^ is a Nordic database within medicine and health.

All databases were searched from inception to February 2021. All translations were by bilingual reviewers.

### Hand Searching of Websites

The following websites were hand-searched by a native speaker: Napha.no, which provides documents from the field of practice in mental health about recovery, peer support work and service user experiences and Erfaringskompetanse.no, which focuses on the lived experiences of service users and carers providing resources like subjective service user and carer experiences, stories, service user surveys, non-fiction literature, reports, poetry, essays and books.

Literature from survivor research, activism and from people with lived experience of mental health problems were searched: All online issues of Asylum Magazine for the past 10 years and the websites of Recovery in the Bin, Mad in America and Madness Canada were hand-searched for documents.

### Google Scholar Searches

Google Scholar was searched for articles. Search terms were: (mental health) AND (narrative OR story) AND (use OR misuse OR opportunities OR criticism OR critique OR possibilities). It was searched until 3 pages in a row resulted in no further relevant documents.

### Backwards and Forwards Citation

Reference lists of all included documents were hand-searched for includable documents. Forwards citation tracking was conducted using Google Scholar for all includable documents.

### Expert Consultation

Once a list of included documents was assembled, eight experts from narrative curation, survivor research, service user involvement and mental health research were asked for missing documents, and then forward and backward citation was conducted.

### Filtering of Documents

References generated by database searches was exported to EndNote, and duplicates removed. A pilot screening of 200 documents was conducted by the lead researcher (CY) and a second researcher (DF) to establish adequate concordance. Pilot documents were screened for title, abstract and full text. The lead researcher and a second researcher (SRE) screened all documents identified from databases. Ten percent of all records were screened by both, and concordance on title, abstract and full text was recorded. Acceptable concordance was ≥90% agreement on exclusions on title or abstract, and 100% was achieved. Acceptable concordance was 100% for inclusion on full text, and was achieved. Documents in Swedish, Danish or Norwegian were screened by a researcher fluent in the language used.

### Data Abstraction

A data abstraction table and guidance were designed and piloted using a convenience sample of 16 documents, and the design was refined. The final data abstraction table (online supplement 2) was piloted with 10 documents. The table included: (a) Citation information: author, year, title, journal, country of lead author, country the research comes from; (b) Characteristics of study: design, number of participants; (c) Uses and misuses of mental health lived experience narratives. For each document, all uses and misuses were recorded. *Uses* integrated items from the list of 13 possible uses of mental health lived experience narrative collections provided in the VOICES typology^17^ where relevant, or inductively extended this list where not. Text from documents concerning uses or misuses was extracted from documents to be analyzed. Open questions were entered in to the DAT as misuses and then later analyzed separately.

### Quality Assessment

No quality assessment was undertaken because this is a conceptual review to map out uses and misuses, rather than to assess the evidence base for uses or misuses.

### Synthesis

Two initial typologies were produced by iteratively combining entries in the DAT relating to (a) uses and (b) misuses. Items in these two typologies were discussed, critically reflected upon and refined by the research team, and names of items were updated. Items were then partitioned into superordinate categories which were identified inductively, and reviewed by the research team. Narrative summaries were developed to describe the contents of included documents that related to each named misuse, to make these contents readily available.

To enhance the robustness of results and due to the possibility that mental health lived experience may offer additional insight into the topic, a sub-group analysis was conducted of documents in which at least one author stated that they had lived experience of mental health problems; through this analysis, the misuses presented in these documents were identified.

The recommendations were developed by the research team based on analysis and discussion of the misuses of lived experience narratives.

### Research Team

The research team brought a range of academic and clinical expertise, including in survivor research; critical qualitative health research; mental health services research; critical disability studies; qualitative research within recovery; participatory methods; and clinical psychology. The team included an information specialist, a statistician and a chief executive of a user-led charity. C.Y., S.R.E., D.F., J.L.B., and J.V. identify as having lived experience of mental health distress, and some have publicly shared lived experience narratives.

## Results

**Fig. 1.**
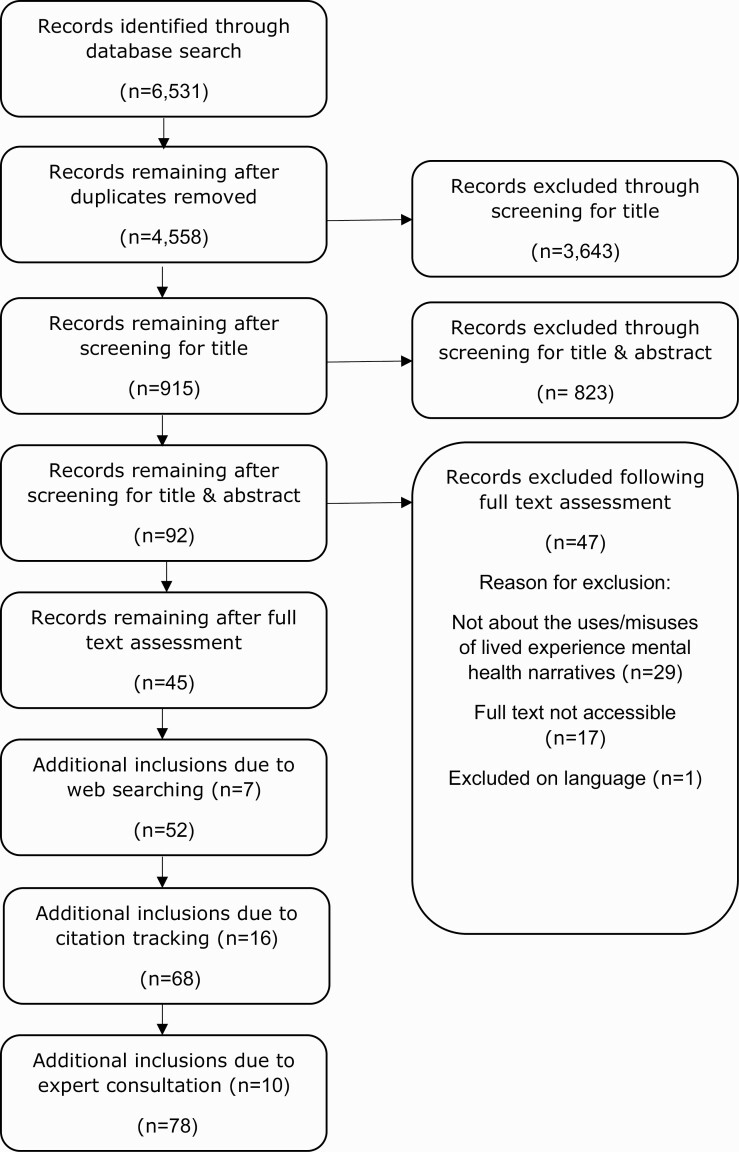
PRISMA flow diagram.

The PRISMA flow diagram for the systematic review is shown in figure 1.

The 78 documents included were from 11, all high-resource, countries: UK (*n* = 27), USA (*n* = 19), Australia (*n* = 13), Norway (*n* = 6), Canada (*n* = 5), Germany (*n* = 2), Ireland (*n* = 2), Belgium, Finland, France, and Sweden (all *n*s = 1). Included documents are listed in online supplement 3. In 30 (38%) documents at least one author stated that they have mental health lived experience.

### Uses of Lived Experience Narratives

Twenty-seven uses of lived experience narratives were identified, which were grouped into five categories shown in table 1.

**Table 1.** Uses of lived experience narratives

Name of use	Actual use *N*	Proposed use *N*	Document ID (actual/proposed)
Political			
Assisting with achieving policy change aims	1	5	70/22, 35, 40, 65, 73
Using the voices of recovery or madness as agents of change	1	4	31/17, 34, 35, 44
Building a narrative collection to act as an evidence base	1	4	58/8, 34,40, 76
Emancipation through having a voice	0	14	13, 14, 17, 18, 20, 24, 27, 34, 40, 44, 45, 56, 64, 70
Recruiting people to a cause	0	1	18
Societal			
Reconceptualising definitions of mental illness	5	14	1, 2, 3, 4, 7/14, 23, 24, 26, 27, 30, 35, 45, 56, 58, 62, 63, 64, 72
Reducing stigma such as in anti-stigma campaigns or apps	2	6	43, 59/1, 9, 22, 51, 57, 70
Encouraging people to seek mental health treatment	1	1	36/9
Using in research activities such as data for analysis	0	9	8, 12, 13, 14, 17, 23, 28, 32, 33
Encouraging others to share their story	0	1	39
Community			
Organizing, peer support and solidarity	1	8	34/17, 18, 24, 27, 30, 31, 44, 45
Opening dialogue between different perspectives	0	4	5, 23, 30, 38
Promoting fundraising activities	0	2	17, 70
Community participation	0	2	74, 77
Using in support groups for shared reading and analysis	0	1	41
Increasing visibility for a specific group, for example, the Black and Minority Ethnic community	0	1	34
Service level			
Improving mental health and social care services	1	11	36/1, 11, 16, 17, 19, 24, 28, 30, 31, 38, 73
Highlighting inhumane or oppressive psychiatric treatment	0	4	7, 11, 45, 71
Developing partnership and helpful relations in services	0	2	75, 78
Developing clinical theory and practice	0	1	14
Evaluating mental health services	0	1	48
Individual			
Using as a therapeutic tool in a digital intervention	5	0	51, 47, 60, 61, 68
Enhancing the personal recovery of curator, narrator and/or recipient	1	10	1/6, 66, 22, 35, 34, 36, 21, 49, 52, 69
Using in therapy sessions with a mental health worker	2	4	21, 25/51, 41, 15, 67
Using for self advocacy for narrators	0	2	8, 18
Using in meetings between peer support workers and service users	0	1	51

### Misuses of Lived Experience Narratives

Eleven misuses were identified, which were grouped into three categories shown in table 2.

**Table 2.** Misuses of lived experience narratives

1. Misuse relating to the narrative	1.1 Narratives may be co-opted
	1.2 Narratives may be used against the author/cause
	1.3 Narratives may be used for different purpose than authorial intent
	1.4 Narratives may be reinterpreted by others
	1.5 Narratives may become patient porn
	1.6 Narratives may lack diversity
2. Misuse relating to the narrator	2.1 Narrator may be subject to unethical editing practises
	2.2 Narrator may be subject to coercion
	2.3 Narrator may be harmed
3. Misuse relating to the audience	3.1 Audience may be triggered
	3.2 Audience may misunderstand

### Misuse Category 1: Relating to the Narrative

#### Narratives may be Co-opted

Co-option as a concept referred to narratives being used as a commodity,^3^ for instance to promote neoliberal agendas:^28^

Mental health service systems have been able to absorb resistance accounts, sanitize them, and carry them forward in ways that are useful for them, without disrupting their dominant practices [#17].^20^

Co-opted narratives can be depoliticized,^29^ edited and censored and used by mental health systems to promote their services^30^:

Storytelling practices now get processed through resiliency and recovery metanarratives that continue to position both the problem and its potential solution at the level of individual bodies [#64].^31^

Sharing of narratives can promote an individualistic pathology approach which focuses on an individual’s responsibility to recover, and hence ignores or marginalizes wider social, political, cultural and economic contextual factors.^3,19^

#### Narratives may be Used Against the Author

The narrative may be used against the author:

There is little denying the power of story… until our own stories get taken from us, positioned against us, and used to determine our value as some sort of human commodity….when those in power take on the telling of the stories of marginalized peoples, storytelling can serve as a weapon that obfuscates the truth, and further entrenches social injustices [#20].^32^

#### *Narratives may be* U*sed for* D*ifferent* P*urpose than* A*uthorial* I*ntent.*

The narrative may be used for different purposes than the author may have intended. Once a narrative is published who owns and controls the use of the narrative?^11^ A narrative could be decontextualized from its original purpose and authorial intention.^33^ Once a narrative has been published in one place it can be used in other ways without the narrator’s consent.^33^ The narrator may agree for their narrative to be used in a research project, however the narrative may be used in other ways such as being published in a newspaper.^30^ There is also the issue of whether withdrawal of narratives is possible and how a narrator may do this.^30^

#### Narratives may be Reinterpreted by Others

The narrator may not be given the space to offer their own interpretation of their narrative.^34^ Narratives can be reduced to data by researchers who do not engage in dialogue with the authors of the narrative and reinterpret their words uncritically perpetuating a physician/patient research dynamic.^33^ The researcher has the power of translation and of interpretation^35^:

The interpretation of people’s stories by researchers may result in the imposition of narrative templates that erase complexities and contribute to the perpetuation of oppression [#26].^36^

There is very little survivor-controlled analysis of individual and collective histories.^37^ Peer researchers are often called upon to share their narrative, but are rarely involved in the analysis of their narratives.^29^ The assignment of the tasks of understanding and making meaning of madness to researchers rather than to those directly concerned results in the great majority of narrative analyses perpetuating the role and power divisions central to psychiatric treatment.^38^ Survivor-controlled research has an aim of minimizing the researcher’s interpretation of narratives.^38^

#### Narratives may Become Patient Porn

Narratives are used by organizations and can become patient porn. By pornographic it is meant that, “while some people reveal their most intimate personal details, others achieve relief through passive watching, while still others profit from the collaboration of those on the front lines in compromised positions [#17].” ^20^ The term porn can be used to examine how stories are told, heard, performed, consumed, valued and how they might be commodified.^19^

#### *Narratives may* L*ack* D*iversity.*

There is a risk of overgeneralization in narratives shared which could give rise to harmful stereotypes.^39^ There is also a danger that certain voices may be excluded, for example, national mental health campaigns tend to use younger voices and photogenic faces, and speakers at academic conferences are often white, middle-class cis women and men.^19^ The issue of intersectionality is a challenge to be addressed wherein the effects of overlapping discrimination and disadvantage due to racial identity, class, gender, sexuality and disability as well as mental health status, may be elided.^33,40^ There are many narratives of healing and recovery, but there needs to be more space for stories of resistance and opposition, collective action and social change.^35^ There is a specific need to include narratives which critique the mental health system.^32^ Narratives promoted by mental health services tend to be “risk free” and are shared in a way which will not cause any distress to recipients.^30^ Narratives can be molded to fit templates that show gratitude to mental health services and do not show complexity or systemic problems.^3^ Narratives which are “inspirational” or “insightful” tend to be included.^3^ Narratives which confuse or challenge tend to be excluded,^3^ as do narratives of nonrecovery and noncompliance.^41^

### Misuse Category 2: Relating to the Narrator

#### Narrator may be Subject to Unethical Editing Practices

The process of editing narratives may not be ethical and narrators may be required by editors or organizations to include or exclude certain details. One example of this is where narrators were given rules regarding what to include in their narrative such as needing to show gratitude to the police.^30^

#### *Narrator may be Subject to* C*oercion.*

People may be coerced into sharing their narratives, such as by the mental health service that they are using. One example of this was where “recovery narratives were treated as a requirement for discharge from inpatient care, creating conditions in which patients were coerced into not only sharing a narrative but one with specific features [#36].” ^30^ Recovery in the Bin believe that “being made to feel like you have to tell your “story” to justify your experience is a form of disempowerment, under the guise of empowerment [#50].” ^42^ Some believe that the imperative to narrate traumatic experiences is another form of oppression.^19^

#### Narrator may be Harmed

Disclosing one’s experiences of mental distress may cause harm to the narrator.^19^ There is potential distress in the process of sharing of a person’s narrative which could cause retraumatisation.^43^ In the process of selecting narratives, certain narratives may be rejected, which may cause harm to the narrator.^30^

### Misuse Category 3: Relating to the Audience

#### Audience may be Triggered

The accessing of a narrative can harm the recipient.^33^ There might be potential negative effects with reading anorexia nervosa narratives, which may be associated with social learning processes of imitation, identification and competition.^44^ Narratives can be triggering to recipients and cause distress,^45^ especially in the case of self-harm.^46^

#### Audience may Misunderstand

There is the challenge of listening to and learning from narratives and audience understanding.^47^ The “illness narratives require an active and reflexive audience who are willing to enter into dialogue with the writer and the story.” ^48^ There are “institutionalised and disciplinary ways of hearing and interpreting a mental health service-user,” ^29^ clouding the way a narrative is heard and understood which may be different from the narrator intention.^31^

### Open Questions

Four open questions were identified, drawing attention to areas of uncertainty around whether something constitutes misuse in table 3.

**Table 3.** Open questions relating to lived experience narratives

Open Questions	Does including a researcher’s personal mental health narrative reduce the credibility of their research?
	Should the confidentiality of narrators be protected?
	Who should profit from narratives?
	How reliable are narratives as evidence?

#### Open Question 1: Does Including a Researcher’s Personal Mental Health Narrative Reduce the Credibility of their Research?

The threat of losing credibility related to the reader and not the writer. The potential impact on credibility arises from the possibility that in finding out about the writer’s mental health, a reader may re-evaluate other unconnected aspects of the writer’s work.^49^

#### Open Question 2: Should the Confidentiality of Narrators be Protected?

When a narrator’s face and voice are published there is the ethical tension of ownership of voice and identification and “the need for confidentiality and non-exploitation of people represented in digital stories.” ^50^ There is a need to ensure consent is agreed and privacy protected while not undermining the autonomy of the narrator.^50^ There is the balance between the choice of the narrator to share their face and identity and the protection of confidentially of people sharing their story. For example, if a person chooses to tell their story non-anonymously on YouTube there is the internet-enabled ability to link this information to the person’s different online profile on LinkedIn.

#### Open Question 3: Who should Profit from Narratives?

There is an ethical question as to whether other people should profit from the use of lived experience narratives. There is the issue of researchers who, “by their very self-reflexivity have discovered how to be really effective at stealing stories for their own academic gain.” ^20^ Whereas the “people who share their stories remain disadvantaged, often unpaid, unequal partners while organisations, professionals, and academics benefit through receiving funding and building a career path on the basis of user involvement.” ^3^

#### Open Question 4: How Reliable are Narratives as Evidence?

Narratives can be persuasive, powerful and can generate empathetic responses which can shape debates, but can they be trusted as forms of evidence?^39^ Can people ever really tell their stories effectively?^51^ To what extent can we trust that people’s narratives faithfully describe “what it was really like?” ^52^ However the key question here is:

The criteria for what constitutes evidence is a central question in the philosophy of science and ought to be of concern to everyone who is involved in any kind of mental health initiative [#32].” ^53^

A total of 14 (93%) of the 15 misuses and open questions were contained in documents with at least one author with lived experience. The exception was the narrator may be subject to unethical editing practises.

## Discussion

The review highlighted 27 uses of lived experience narratives divided into 5 categories; political, societal, community, service level and individual. There was some overlap between some of these categories. The review also highlighted 11 misuses of lived experience narratives in three categories; misuses relating to the narrative, misuses relating to the narrator and misuses relating to the audience and highlighted 4 open questions.

### Strengths and Limitations

Strengths were the broad inclusion criteria leading to a large number of papers included, multiple languages and the use of a wide range of publication databases and gray literature sources, including survivor and activist websites such as Recovery in the Bin^42^ and Mad in America.^54^ Another strength was the diversity of the review team and being led by researchers with lived experience of mental distress and sharing their narrative.

A limitation of the review is that it only included publicly available documents where full text was accessible. There was no “right to reply” from organisations identified in included documents as misusing narratives, and further research to obtain their perspectives might illuminate the complexity of some components of the review.

### Recommendations

On the basis of this systematic review, we make seven recommendations for best practice in using recorded mental health recorded narratives in healthcare and community settings.

#### Recommendation 1: Narratives must be Experientially and Representationally Diverse

It is imperative that service users invited to produce narratives in research, education, policy, service, practice, and popular media settings actually reflect those that encounter mental health systems. Thus, those that are seeking out such stories must make a concerted effort to privilege the narratives of BAME, Queer and others whose narratives are often subjugated. Further, narratives should be encouraged to be complex, including those that express resistance, critique, non-compliance and more. This means that those who hold power to solicit, curate, and shape these narratives must resist whatever comfort, complacency, and interests they hold to ensure that a diversity of experiences of both mental health and mental health system encounters are profiled and proliferate.

#### Recommendation 2: Decenter Pathological Logics, and Build Pathways for Ethical Listening

As the intent of the storyteller is processed through an audience, content is always subject to reinterpretation. No two people interpret art alike. Yet, given the dominance of mental health metanarratives as informed through medical and psychiatric powers and a lack of alternative counternarratives circulating in mainstream culture, it is exceedingly difficult to listen to and interpret service user narratives beyond the confines of pathological mental illness/recovery frameworks. To fully address the issues identified in this review, including how service user stories may be misunderstood or reinterpreted by others, requires a cultural shift. This entails long-term systemic socio-cultural work that has been advanced by other social movement activist groups (BAME, Indigenous, LGBTQ2S+) who have demanded their stories be heard on their own terms and through their own sets of cultural values.^55–57^ Change requires first identifying and articulating the issue, and this is clearly done in this review. To help move our listening practices forward Baylosis^58^ offers practical considerations and tools for those working in mental health systems to help us work towards an “ethics of listening” that promotes reflexive listening and work towards honoring and valuing narratives on their own terms.

#### Recommendation 3: Mitigate Harm, Promote Safety

Rarely does an author have control over their own narration: it is almost always intervened on. Yet, service users have particular considerations to content with when storytelling. These include the fact that people, including landlords, employers, family, and friends may hold discriminatory views towards mental health service users. Sharing their stories can lead to long-term consequences, including housing and employment insecurity. Service users are often encouraged by mental health service systems to share their narratives as a part of anti-stigma work. While this choice is framed as up to the service user, often what isn’t recognized is that many marginalized service users have limited choice in their decision-making,^59^ as exemplified by instances of coercion as detailed in this paper. Further, many academic or service provider events are now filmed and posted online. Service user narratives are thus being consumed beyond the scope of the in-room audience, subject to viewing and comments by anyone.

Given that stories are increasingly consumed and shared online, and that they may be easily accessible forever, these risks should weigh heavily. When approaching service users to share narratives as part of your event/agenda, empower them to say no by discussing these risks. Then, if you plan on recording the event, tell the service user when you invite them to storytell, as this may impact their decision. Or, offer to not record their segment, and normalize this practice. Encourage them to use a pseudonym, and discuss why. Consider not tagging their name to videos posted online.

#### Recommendation 4: Recognize the Value of Narratives and Offer Proper Compensation

In broader contexts, individual experiences and narratives are often turned into commodities for profit. Usually, both those telling the story and those harnessing and selling the story recognize this and efforts for equal exchange/remuneration discussed. Yet in the case of service user narratives, rarely do those seeking service user narrators have frank open discussion with them about how these narratives are harnessed for research, promotional, or public relation campaigns, and that there is a monetary value attached to narratives that benefits those soliciting them. Rather, the benefit of sharing narratives is pitched to the service user as to help themselves or others like them. Given the lack of recognition and acknowledgement that there are industries that rely on these narratives and an unequal profit margin, the articulated concern of stealing stories is real. One way to address the issue of commodifying narratives is actively and openly recognizing the monetary value of these narratives, and paying those who offer this commodity a fair and appropriate compensation beyond the token honorarium.

#### Recommendation 5: Research Processes into Lived Experience Narratives should Integrate Lived Experience Researchers

The potential misuse of lived experience narratives in research was raised. The process of the lived experience narrative as a piece of data for non-peer researchers to analyze was criticized and it was recommended to increase the amount of lived experience led analysis.^34^ Of the 15 misuses identified in this systematic review, 14 (93%) of those documents had at least one author with lived experience. This suggests that having people with lived experience as part of the authorship team may contribute to fostering knowledge production that might otherwise be overlooked.

#### Recommendation 6: Consider Imbalances of Power Whenever Using Lived Experience Narratives

Power is a key issue relating to the uses and misuses of lived experience narratives. Foucault stated that “instead of conceptualizing psychiatric power in terms of institutions, with their regularities and rules, one has to understand psychiatric practice in terms of “imbalances of power” with the tactical uses of “networks, currents, relays, points of support, differences of potential” that characterize a form of power”.^60^ In relation to the use of narratives one can consider different people and institutions involved in the process and the imbalances of power with the narrator as service user or survivor typically having the least amount of power. This power imbalance extends to academic life, whereby service users are engaged by “non-user academics motivated more by the exigencies of their own academic advancement than the deeper and more meaningful rebalancing of power and expertise.” ^61^ Non-users need to contend then with their own power in these encounters with narratives and how to use them in the interests of dispersing power to those who hold them.

#### Recommendation 7: Publication Guidelines for Lived Experience Narratives should Include a Description of How Misuses can be Avoided

Editors of journals which include lived experience narratives in their publications could include in their publication guidelines recommendations on their use to prevent misuse and give guidance on for instance improving diversity of lived experience narratives selected for publication. Similarly, curators of book or online collections of lived experience narratives may be guided to avoid possible misuses such as unethical editing practices.

A specific component to include in publication guidelines is the possible dangers of a published lived experience narrative being co-opted or used for other purposes from their authorial intent. Potential approaches to reducing this danger include publishing the narrative using a Creative Commons Attribution Licence specifying the permitted amount of re-use or modification, ensuring (e.g. through a consent process) that narrators are aware of the potential for wider circulation of their narrative, providing guidance on anonymizing narratives, and having clear withdrawal processes. In addition there are a number of “telling your story” guides,^62^ which could be signposted to possible contributors.

## Conclusion

Future work could use the recommendations to establish good practice guidelines for the use of lived experience narratives which could be used by curators, editors, activists, researchers, mental health workers and those wishing to share their narrative.

## Supplementary Material

sbab097_suppl_Supplementary_Material_1

sbab097_suppl_Supplementary_Material_2

sbab097_suppl_Supplementary_Material_3
