# Supplementary material for: Uses and Misuses of Recorded Mental Health Lived Experience Narratives in Healthcare and Community Settings: Systematic Review
Source: Schizophr Bull. 2021 Aug 23;48(1):134–44. doi: 10.1093/schbul/sbab097 (PMC8781345; doi:10.1093/schbul/sbab097)
Supplement: sbab097_suppl_Supplementary_Material_1 [file sbab097_suppl_supplementary_material_1.doc]

Search terms used in PsycINFO database search

| **Number** | **Clause** |
| --- | --- |
| 1 | ("psychological health" or "psychiatric illness" or "psychiatric problem" or "psychiatric disorder" or "mental health" or madness or mad) |
| 2 | ((psychological or mental or emotional) adj distress) |
| 3 | Mental Health/ |
| 4 | 1 or 2 or 3 |
| 5 | (narrative or story or stories or storytelling or telling or tale* or restory or re-story or counternarrative or counter-narrative or memoir* or testimon* or biograph* or autobiograph* or auto-ethnograph* or photovoice) |
| 6 | Biography/ or Autobiography/ or Narratives/ |
| 7 | 5 or 6 |
| 8 | (uses or misuses or opportunit* or criticism* or critique* or possibilit*) |
| 9 | 4 and 7 and 8 |
